# Pathological Subtrochanteric Femoral Fracture as the Initial Presentation of Metastatic Thyroid Carcinoma in a Young Adult: A Diagnostic Challenge

**DOI:** 10.7759/cureus.105431

**Published:** 2026-03-18

**Authors:** Abhishek K Singh, Tobi Oputa, Muhammad Z Tariq

**Affiliations:** 1 Trauma and Orthopaedics, The Royal Oldham Hospital, Manchester, GBR

**Keywords:** cephalomedullary nail, low-energy fracture, metastatic bone disease(mbd), metastatic cancer of unknown primary, metastatic follicular thyroid carcinoma, metastatic thyroid cancer, orthopaedic oncology, pathological fracture, subtrochanteric femur fracture

## Abstract

Pathological fractures should be suspected in young adults presenting with low-energy femoral fractures. We report a 39-year-old woman who sustained a subtrochanteric fracture after trivial trauma while exiting a car. Imaging demonstrated multiple lytic lesions involving the femur and pelvis, with pulmonary nodules suspicious for metastases. Laboratory evaluation showed biochemical hyperthyroidism. Intramedullary fixation was performed after discussion with the Oncology Multidisciplinary Team (MDT), and histopathology of femoral reamings confirmed metastatic thyroid carcinoma, likely follicular type. Thyroid ultrasound showed no suspicious nodules, highlighting discordance between imaging and histology. This case emphasises the importance of considering pathological fracture in atypical presentations, performing oncologic evaluation before definitive fixation, and relying on tissue diagnosis when imaging is inconclusive.

## Introduction

Subtrochanteric femoral fractures typically occur after high-energy trauma in younger individuals or low-energy trauma in elderly patients with osteoporosis. In young adults, fractures following trivial trauma are uncommon and should raise suspicion for underlying pathology such as metastatic disease, primary bone tumours, or metabolic bone disorders. Failure to recognise a pathological fracture may result in inappropriate fixation and compromised oncologic outcomes. Skeletal metastases frequently involve the femur and pelvis and may be the first manifestation of an occult malignancy. Early identification is therefore essential for appropriate surgical planning and multidisciplinary management. This case highlights a diagnostic challenge in which a pathological fracture was the initial presentation of metastatic thyroid carcinoma despite negative thyroid imaging [1-3].

## Case presentation

A 39-year-old woman presented to the emergency department with acute right hip and thigh pain after experiencing a sudden giving way while getting out of a car. There was no history of high-energy trauma, fall from height, or preceding significant injury. She was previously independent and had no known history of malignancy.

On examination, the patient was unable to weight bear. The right lower limb was shortened and externally rotated, with significant pain on attempted movement. Distal neurovascular status was intact. 

Her past medical history included a partial thyroidectomy approximately 20 years earlier for hyperthyroidism, performed overseas. She was not on long-term corticosteroids and had no known metabolic bone disease.

**Figure 1:**
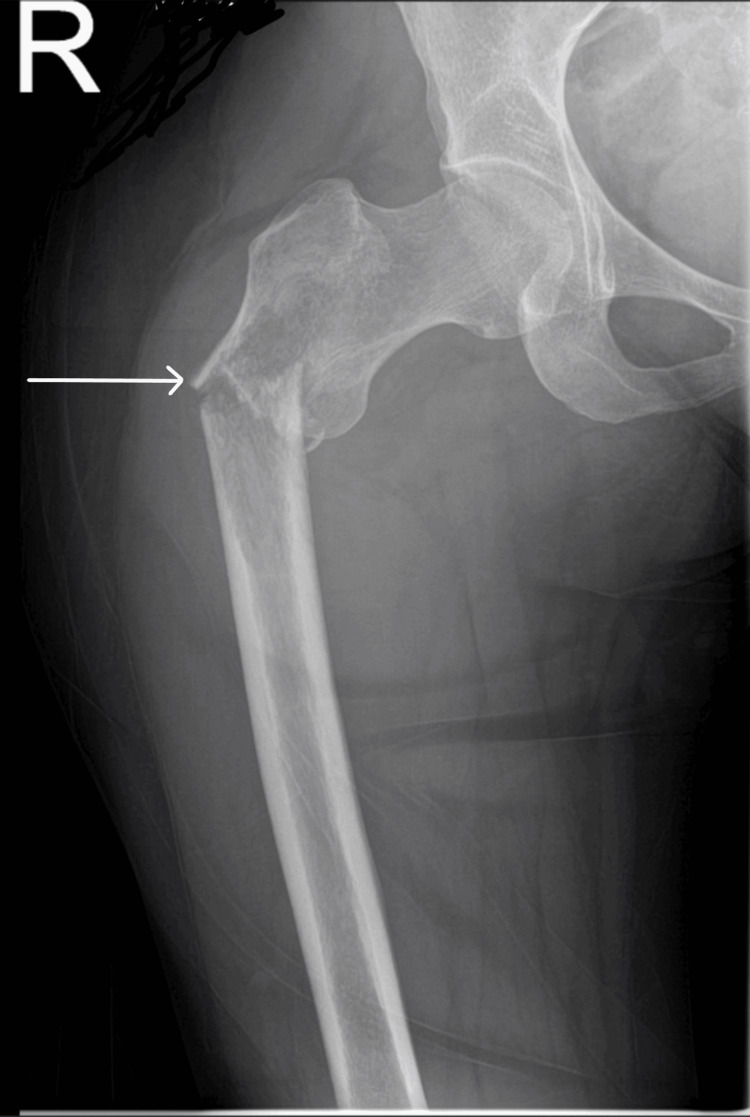
Radiograph of the right hip showing a suspicious lytic lesion

**Figure 2:**
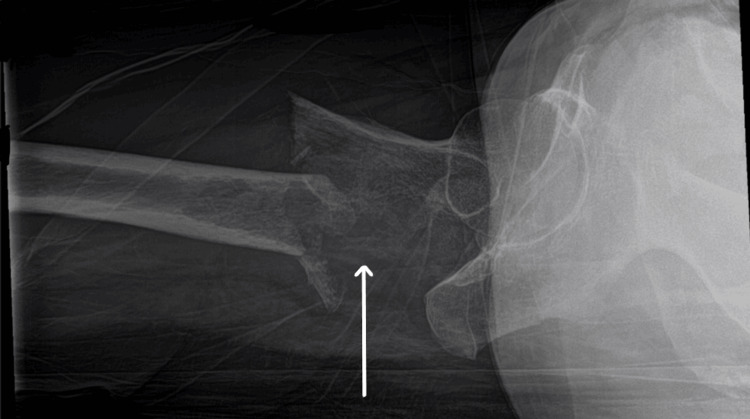
Lateral view of the right hip showing a lytic lesion

Plain radiographs demonstrated a subtrochanteric fracture of the right femur with a suspicious lytic lesion (Figures 1-2). Given the trivial mechanism and atypical fracture pattern, further evaluation for a pathological fracture was initiated.

**Figure 3:**
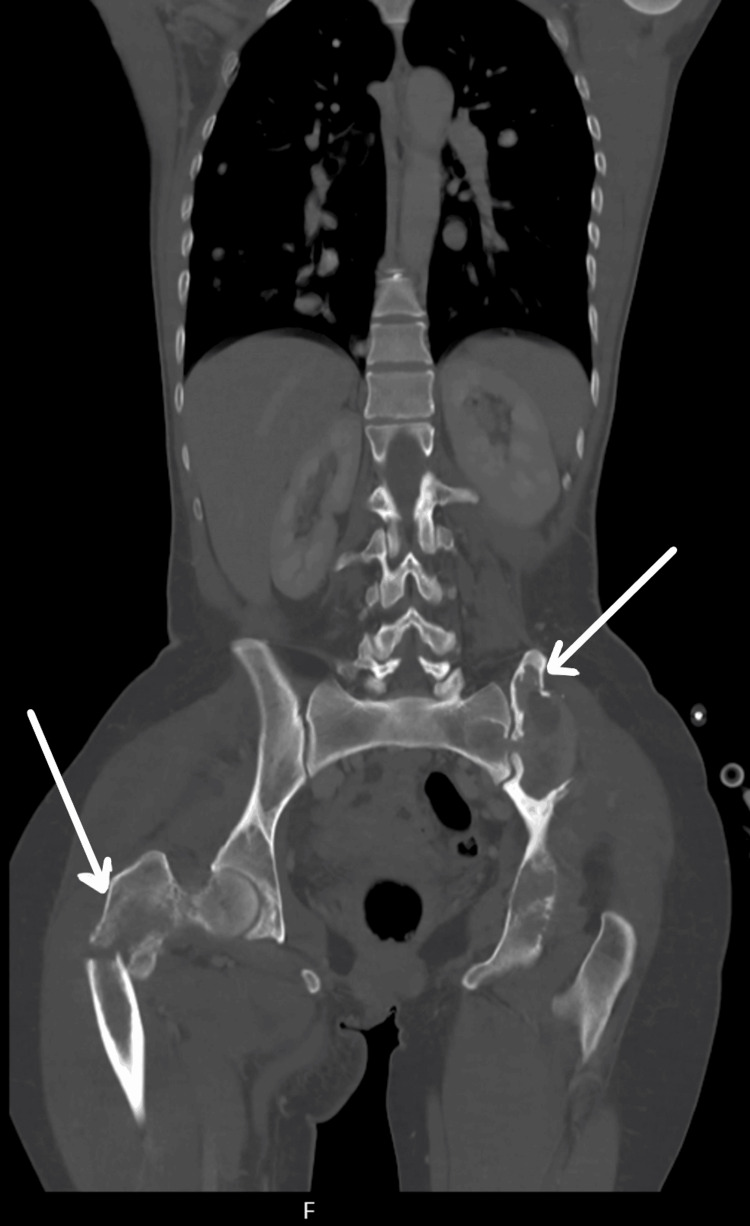
Computed tomography of the thorax, abdomen, and pelvis showing a lytic lesion in the right proximal femur alongside a left pelvic lesion

Computed tomography (CT) of the thorax, abdomen, and pelvis demonstrated widespread bilateral pulmonary nodules suspicious for metastatic disease, along with multiple destructive lytic lesions involving the pelvis, sacrum, lumbar spine, and right proximal femur (Figure 3).

Magnetic resonance imaging (MRI) of the right hip showed a pathological subtrochanteric fracture associated with a large soft tissue mass and extensive marrow infiltration, highly suggestive of metastatic disease. MRI of the brain was unremarkable. Ultrasound of the thyroid demonstrated no suspicious nodules. 

**Figure 4:**
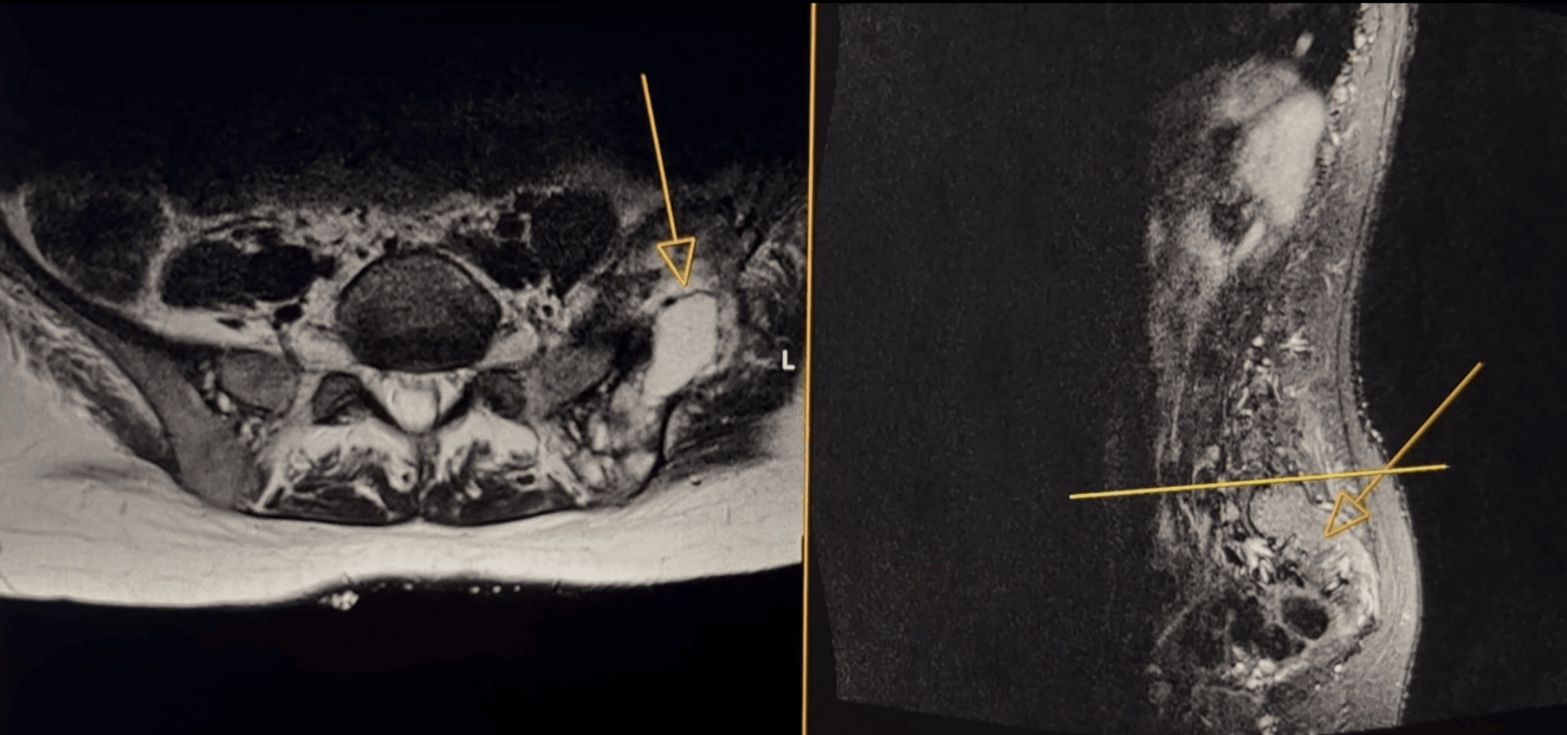
Magnetic resonance imaging of the spine and pelvis confirming soft tissue infiltration and a lytic lesion in the left pelvis

Contralateral pelvic lesions were reviewed in an orthopaedic oncology multidisciplinary team (MDT) meeting. Fracture risk assessment did not meet criteria for prophylactic fixation at that stage; therefore, surveillance and oncologic referral were advised.

Given the high Free T4 and T3 and low levels of thyroid-stimulating hormone (TSH), this indicated evidence of thyrotoxicosis, and the patient was medically optimised with propranolol and carbimazole perioperatively to avoid surgical stress-related thyroid storm (Table 1).

**Table 1:** Laboratory results Key findings included a mildly elevated CA 19-9 level (41 U/mL), elevated inflammatory markers, and biochemical hyperthyroidism (T3 15.82 pmol/L; T4 31.5 pmol/L; TSH 0.01 mIU/L). Renal function, liver function tests, calcium, bone profile, and myeloma screening were within normal limits. CA 19-9: cancer antigen 19-9; CRP: C-reactive protein; ALP: alkaline phosphatase; TSH: thyroid-stimulating hormone

Parameter	Result	Reference range
CA 19-9	41 U/mL	0-37 U/mL
CRP	60.6 mg/L	0-9.9 mg/L
Calcium (adjusted)	2.39 mmol/L	2.20-2.60 mmol/L
ALP	70 U/L	30-130 U/L
TSH	0.01 mIU/L	0.4-4.0 mIU/L
Free T4	31.5 pmol/L	10-22 pmol/L
Free T3	15.82 pmol/L	3.1-6.8 pmol/L
Serum electrophoresis	Normal	-
Free light chains	Normal	-

Surgical management

The patient underwent intramedullary fixation with a cephalomedullary nail. Intraoperatively, tumour tissue was encountered at the fracture site, and reamings were sent for histopathology.

Histopathology

Microscopy showed thyroid follicles containing colloid with macro- and microfollicular architecture, haemorrhage, and necrosis. Immunohistochemistry demonstrated positivity for TTF-1, PAX8, thyroglobulin, and CK7 and negativity for CK20. These findings confirmed metastatic thyroid carcinoma (likely follicular subtype).

Postoperative course

The patient recovered uneventfully from surgery and was referred to endocrinology and oncology services for systemic management and further staging.

## Discussion

Low-energy subtrochanteric femoral fractures in young adults are uncommon and should be approached as pathological until proven otherwise. UK guidance emphasises that fractures occurring with minimal trauma in non-osteoporotic patients warrant a structured pathway to exclude metastatic bone disease [4].

Early recognition is crucial, as premature fixation without adequate staging may compromise oncological management. In this case, prompt escalation to advanced imaging identified disseminated metastatic disease. However, this case was a diagnostic challenge in particular as the initial imaging and sonography were unable to detect the primary cancer source. Only after histology was the primary cancer confirmed. A retrospective cohort study showed that at the initial presentation of metastatic follicular carcinoma, the predominant metastatic site was the bone [5].

Recent literature highlights the importance of obtaining tissue diagnosis, even in fractures that appear metastatic on imaging, due to the risk of primary bone sarcoma or unexpected malignancy [6].

Tumour markers such as cancer antigen 19-9 (CA 19-9) may support assessment but are non-specific and should not be used in isolation to identify a primary malignancy [7]. The clinical picture in this patient aligned with a cancer of unknown primary presentation, for which European Society for Medical Oncology (ESMO) guidelines emphasise histology, immunohistochemistry, and targeted investigation rather than broad non-specific testing [8].

From an orthopaedic perspective, intramedullary fixation is an appropriate stabilisation strategy in selected cases of proximal femoral metastatic disease, enabling early mobilisation and pain control when combined with tissue sampling and MDT input [9].

## Conclusions

Low-energy subtrochanteric femoral fractures in young adults are rare and should be considered pathological until proven otherwise. This case underscores the importance of maintaining a high index of suspicion, performing comprehensive radiological, histopathological, and laboratory evaluation, and involving specialist orthopaedic oncology services early. A structured multidisciplinary approach allows safe fracture stabilisation while ensuring timely histological diagnosis and appropriate oncological management.

## References

[1] Bhandari M, Swiontkowski M (2017). Management of acute hip fracture. N Engl J Med.

[2] (2022). British Orthopaedic Association. BOAST: Management of Metastatic Bone Disease. London: British Orthopaedic Association. BOASt - Management of Metastatic Bone Disease.

[3] Trompeter A (2022). Management of metastatic bone disease (MBD). Injury.

[4] Parameswaran R, Shulin Hu J, Min En N, Tan WB, Yuan NK (2017). Patterns of metastasis in follicular thyroid carcinoma and the difference between early and delayed presentation. Ann R Coll Surg Engl.

[5] Lin JD, Huang MJ, Juang JH (1999). Factors related to the survival of papillary and follicular thyroid carcinoma patients with distant metastases. Thyroid.

[6] Verspoor FG, Hannink G, Parry M, Jeys L, Stevenson JD (2023). The importance of awaiting biopsy results in solitary pathological proximal femoral fractures: do we need to biopsy solitary pathological fractures?. Ann Surg Oncol.

[7] Lee T, Teng TZ, Shelat VG (2020). Carbohydrate antigen 19-9 - tumor marker: past, present, and future. World J Gastrointest Surg.

[8] Mirels H (1989). Metastatic disease in long bones. A proposed scoring system for diagnosing impending pathologic fractures. Clin Orthop Relat Res.

[9] Lai YS, Chuang SH, Kuo YJ, Cheng SJ, Chen YP (2025). Comparative outcomes of internal fixation versus prosthetic reconstruction in the treatment of proximal femoral metastases: a systematic review and meta-analysis. EFORT Open Rev.

